# Supplementary figures and images for: A Transcriptomic Analysis of Xylan Mutants Does Not Support the Existence of a Secondary Cell Wall Integrity System in Arabidopsis
Source: Front Plant Sci. 2018 Mar 27;9:384. doi: 10.3389/fpls.2018.00384 (PMC5881139; doi:10.3389/fpls.2018.00384)

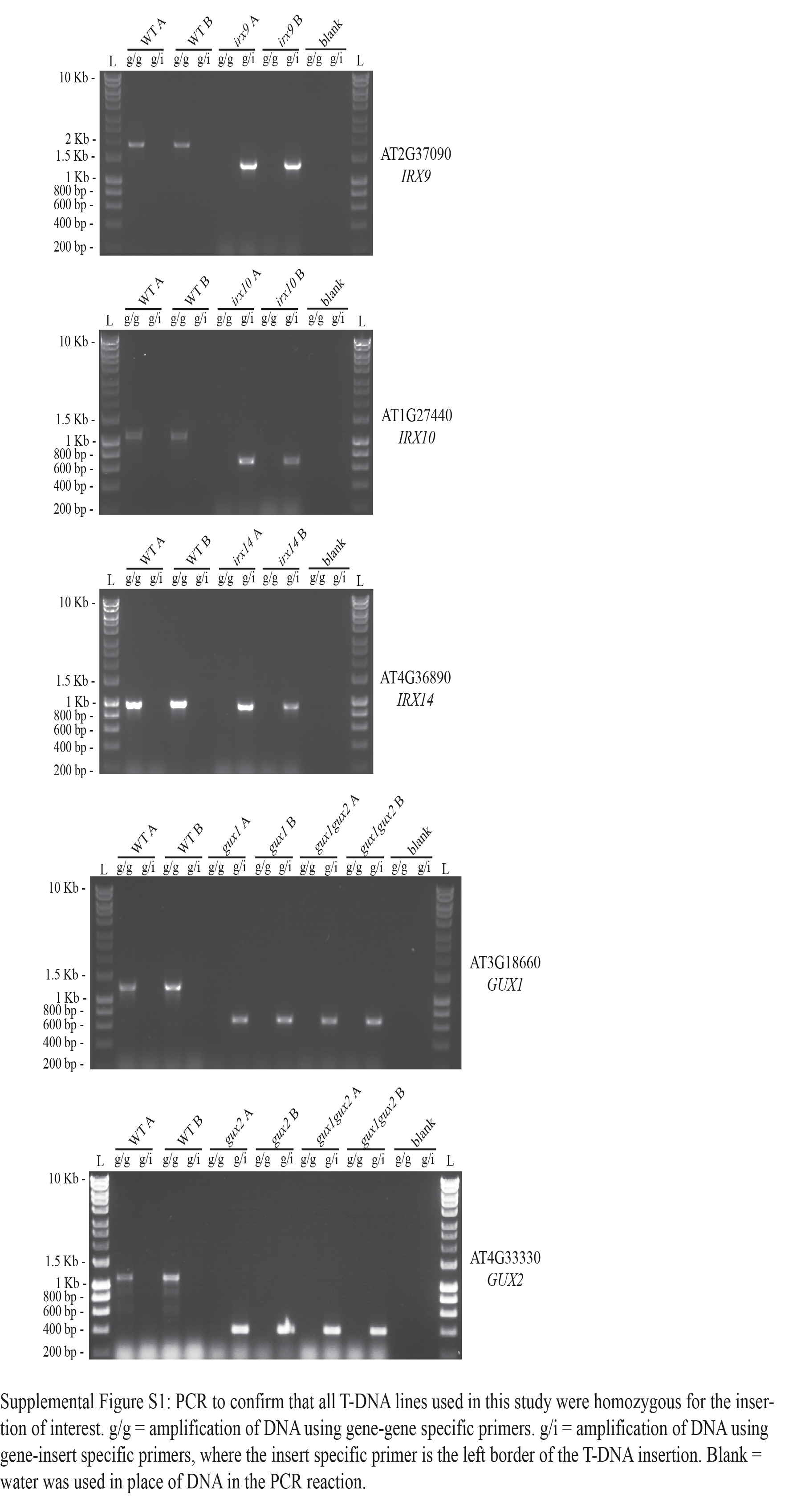

Supplement: Supplemental Figure 1 — PCR to confirm that all T-DNA lines used in this study were homozygous for the insertion of interest. g/g = amplification of DNA using gene-gene specific primers. g/i = amplification of DNA using gene-insert specific primers, where the insert specific primer is the left border of the T-DNA insertion. Blank = water was used in place of DNA in the PCR reaction. [file Image1.TIF]

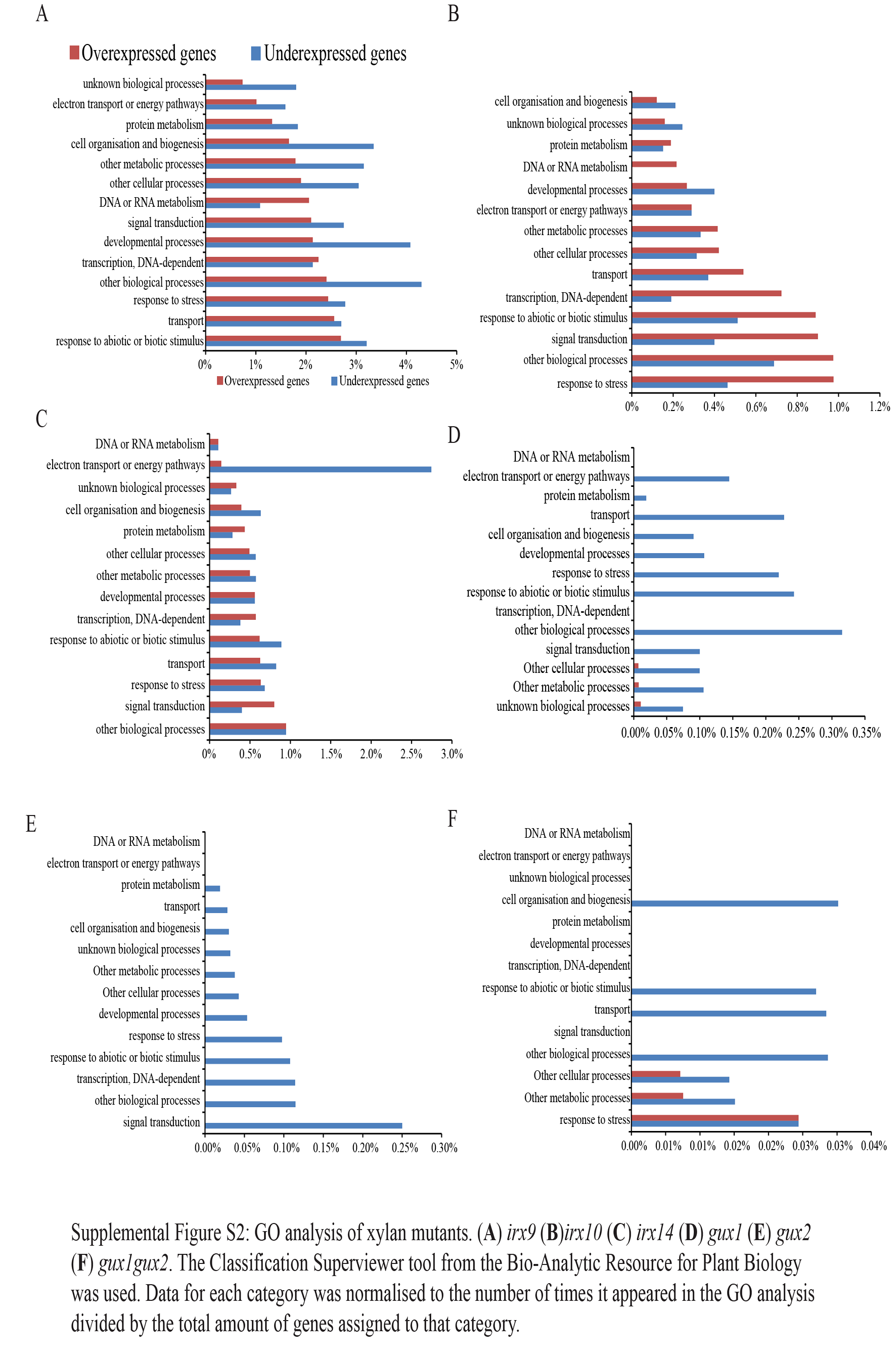

Supplement: Supplemental Figure 2 — GO analysis of xylan mutants. (A) irx9 (B) irx10 (C) irx14 (D) gux1 (E) gux2 (F) gux1gux2. The Classification Superviewer tool from the Bio-Analytic Resource for Plant Biology was used. Data for each category was normalized to the number of times it appeared in the GO analysis divided by the total number of genes assigned to that category. [file Image2.TIF]
